# Exploring Mental Functions in Deafblindness: A Multi‐Method Analysis Using the International Classification of Functioning, Disability, and Health framework

**DOI:** 10.1002/alz70860_099148

**Published:** 2025-12-23

**Authors:** Shirley Dumassais, Walter Wittich

**Affiliations:** ^1^ École d'optométrie, Université de Montréal, Montreal, QC, Canada; ^2^ École d'optométrie, Université de Montréal, Montréal, QC, Canada

## Abstract

**Background:**

Individuals living with deafblindness can face complex cognitive and psychological challenges that can significantly affect their daily functioning, independence, and overall quality of life. Despite these challenges, there is a notable lack of standardized cognitive assessments that are both accessible and suitable for this population, limiting the ability to accurately assess their cognitive and psychological health. Developing such measures requires a foundational understanding of which aspects of cognitive and psychological functioning are most affected by deafblindness. To address this gap, this study aimed to identify and categorize core mental functions relevant to individuals with deafblindness using the International Classification of Functioning, Disability, and Health (ICF) framework.

**Method:**

A multi‐method approach was used, integrating a systematic literature review, an expert survey, and a qualitative study. The systematic review included 147 studies, from which 314 outcome measures were identified. The international expert survey involved 105 professionals who answered an online survey. The international qualitative study consisted of online interviews and focus groups with 72 individuals with deafblindness and/or their caregivers who shared their lived experiences. Identified meaningful concepts from the collected data were linked to ICF categories.

**Result:**

Commonly identified functions across all three research methods included (b114) orientation, (b117) intellectual functions, (b126) temperament and personality, (b130) energy and drive, (b134) sleep, (b147) psychomotor functions, (b152) emotional functions, (b156) perceptual functions, (b164) higher‐level cognitive functions, (b167) mental functions of language, and (b180) experience of self and time. The systematic literature review and the qualitative study additionally highlighted (b140) attention, (b144) memory and (b160) thought functions.

**Conclusion:**

Integrating lived experience, literature, and expert survey data identified consistent mental function categories relevant to deafblindness, supporting the ICF framework's applicability for standardizing functional assessments in research and clinical settings while building the basis for the development of standardized cognitive assessment priorities for international use. The literature review provided insight into existing including individuals with deafblindness. The expert survey captured professional insights on observed client experiences and areas for improvement in practice. The qualitative study offered a detailed exploration of how cognitive and psychological health is affected by deafblindness from the lived experience perspective.

